# Supplementary figures and images for: SRS2 is required for MUS81-dependent CO formation in zmm mutants
Source: PLoS Genet. 2025 Aug 7;21(8):e1011637. doi: 10.1371/journal.pgen.1011637 (PMC12349706; doi:10.1371/journal.pgen.1011637)

A

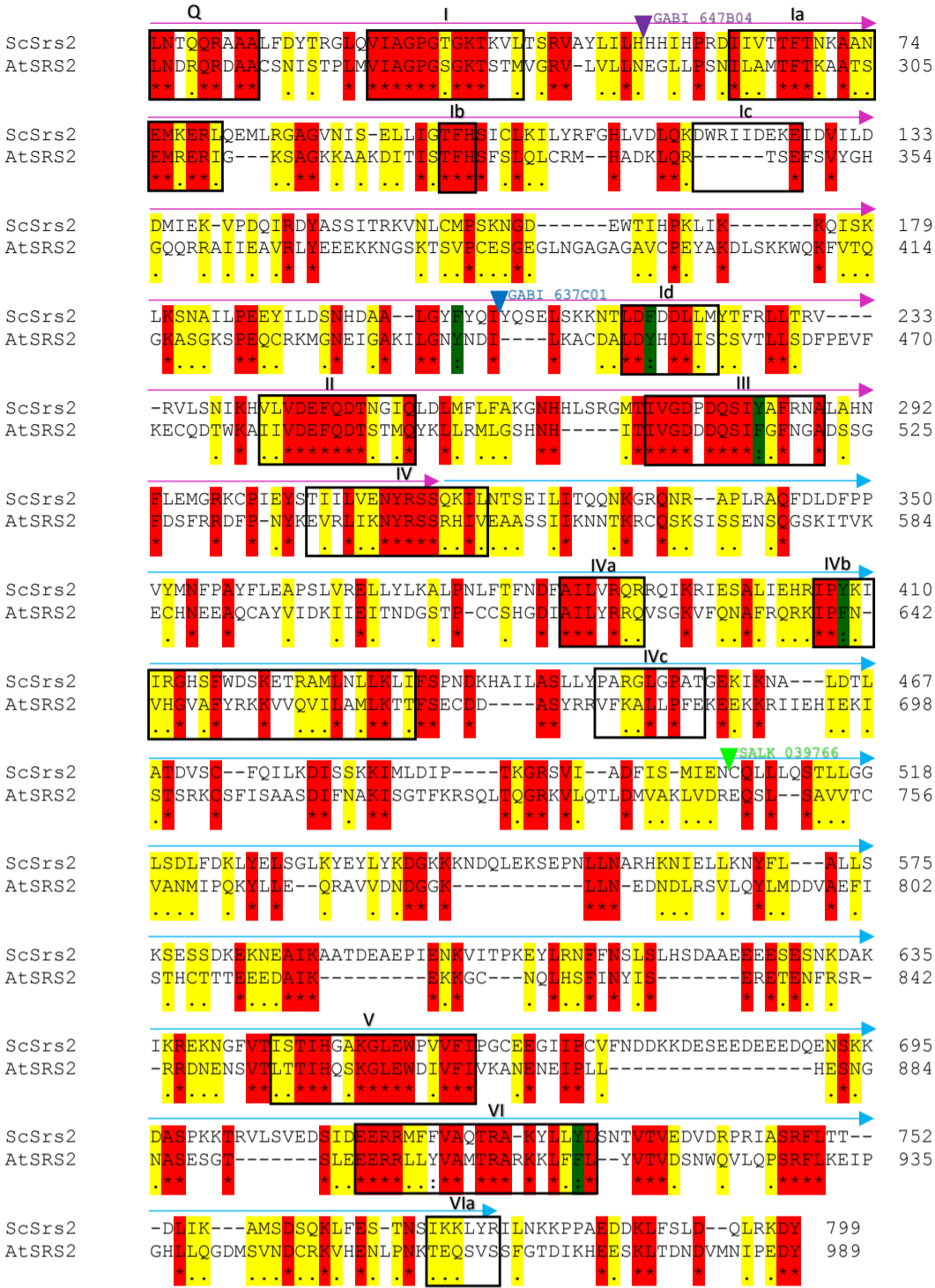

B

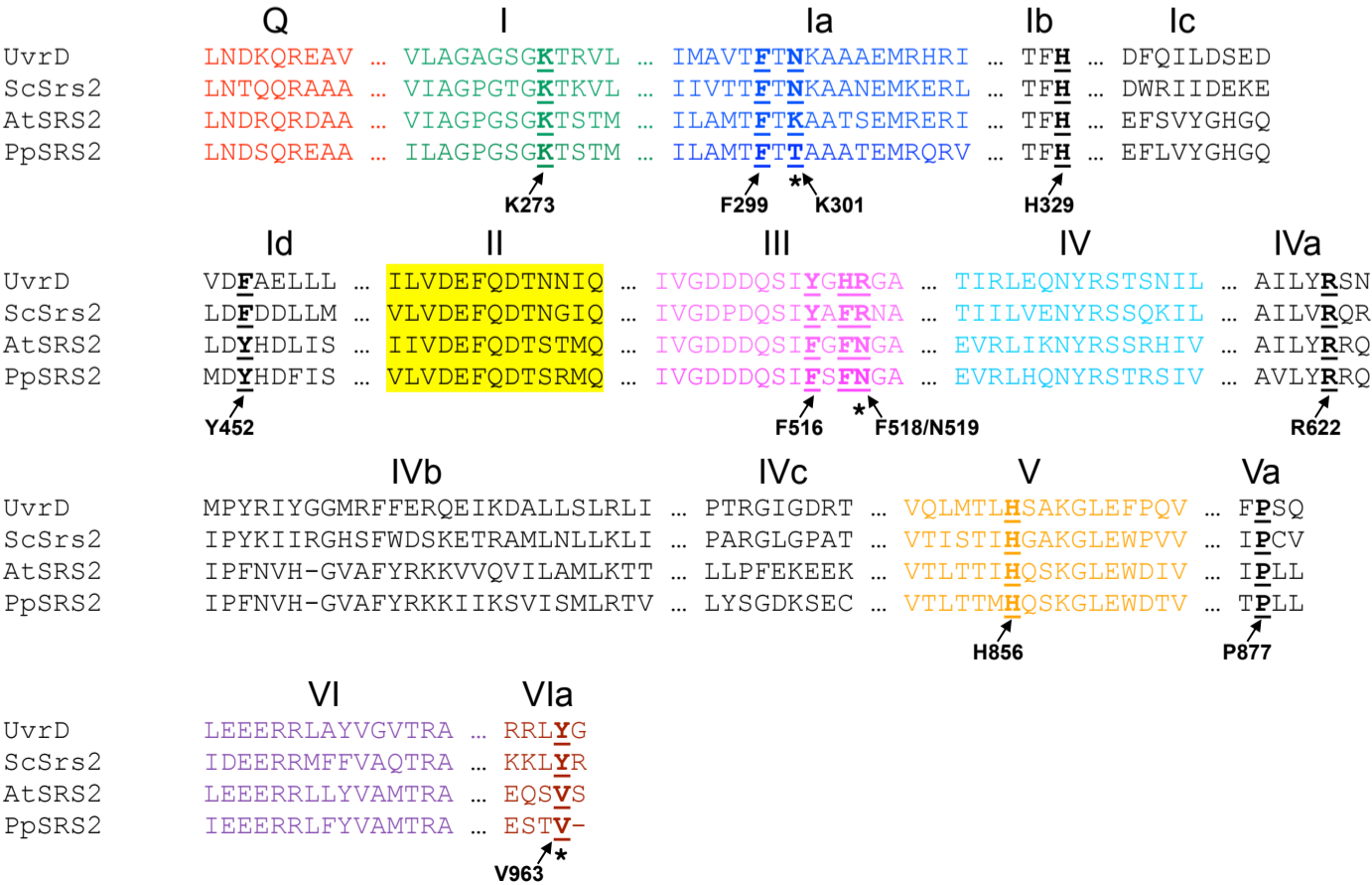

Supplement: S1 Fig — (A) Identical (red), conserved (green) and semi-conserved (yellow) amino acids are depicted. Magenta and turquoise arrows show UvrD-like helicase ATP-binding and UvrD-like helicase C-terminal domains, respectively. Identity 26,26% (219/ 834), similarity 43,53% (363/ 834). (B) Alignment of the helicases domain protein sequences of UvrD, yeast Srs2, A. thaliana SRS2, and Physcomitrella patens SRS2. Comparison of the amino acid residues of the different conserved helicase domains shown to be essential for ScSrs2 function are underlined in bold. Three of these amino acids are not conserved (neither identical nor similar) in Arabidopsis and P. patens and are marked with an asterisk. The amino acids indicated by arrows correspond to those of Arabidopsis. Y775 in yeast corresponds to V963 in Arabidopsis and V1120 in P. patens. (PDF) [file pgen.1011637.s001.pdf]

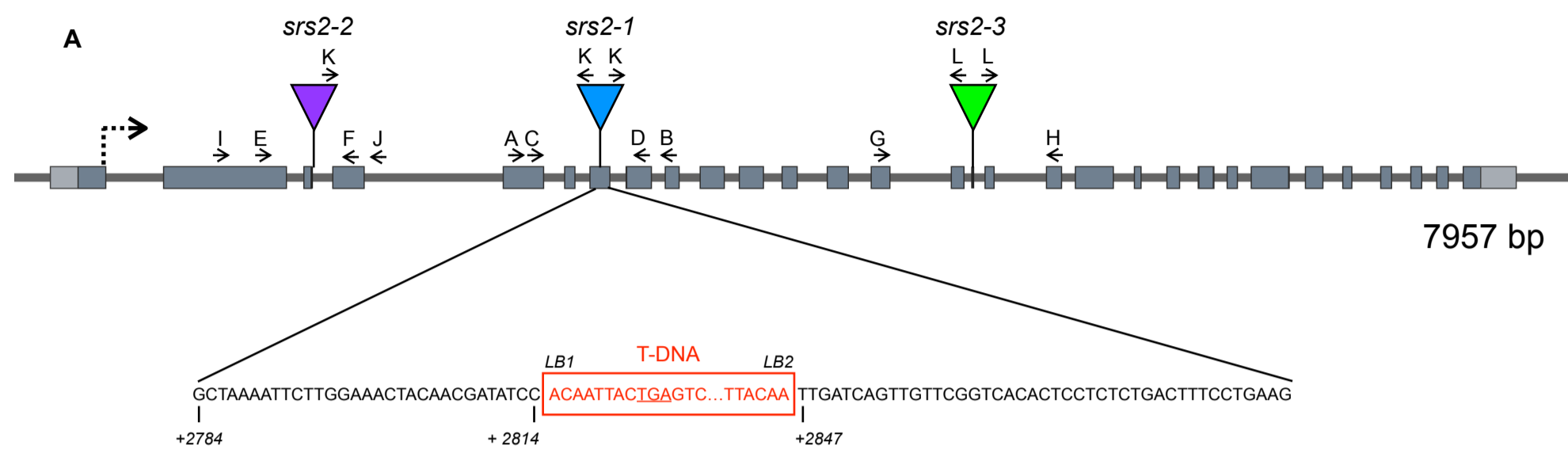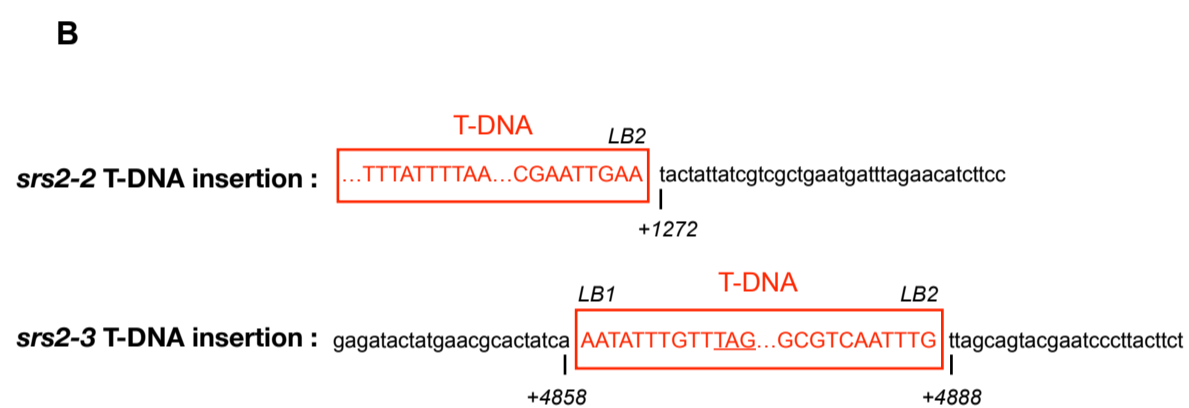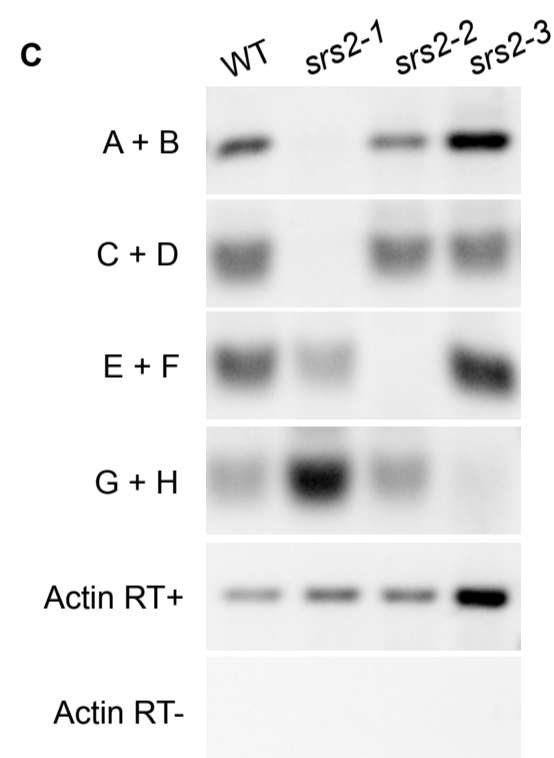

Supplement: S2 Fig — (A) Structure of SRS2 and T-DNA insertion mutant alleles. Dark grey boxes show exons and light grey boxes indicate 5’ and 3’ untranslated regions. The position of the T-DNA insertions is indicated (purple, blue, or green triangles) with arrows showing the orientation of the left border and sequences of the T-DNA/chromosome junctions below (SRS2 sequence in black and T-DNA sequence in red). In srs2–1, insertion is accompanied by a 32 bp deletion in exon 7. A putative in-frame TGA codon is underlined. Numbering under the sequences is relative to the SRS2 start codon. (B) Sequences of the T-DNA/ chromosome junctions in srs2–2 and srs2–3. Putative in-frame stop codon is underlined. Numbering under the sequences is relative to the SRS2 start codon. (C) RT-PCR analyses of transcripts of srs2 insertion mutants. Amplification of the actin transcript (Actin RT+) was used as a control for RT-PCR. The positions and orientations of the PCR primers are shown with capital letters in the diagram. (PDF) [file pgen.1011637.s002.pdf]

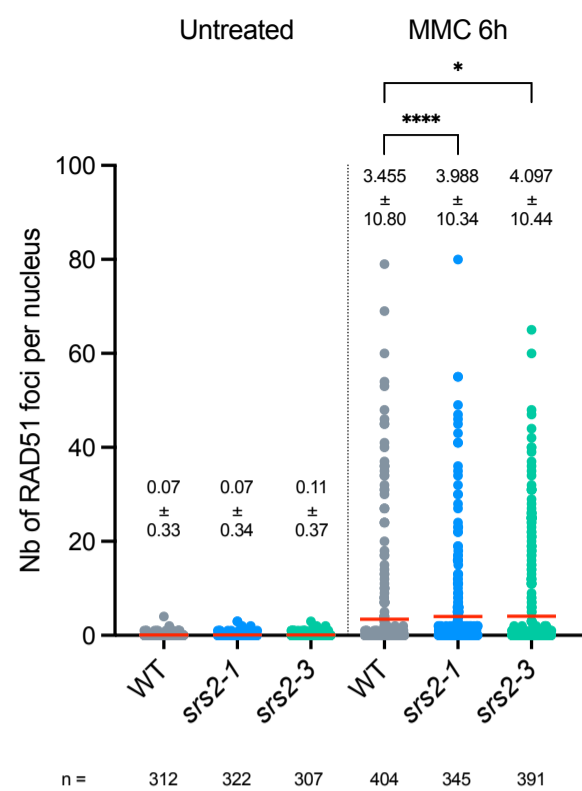

Supplement: S3 Fig — Quantification of RAD51 foci in root tip nuclei of WT, srs2–1, and srs2–3 mutant lines before and after MMC treatment. Data are shown as mean ± SD, with n indicating the number of cells analyzed. More than 300 nuclei from at least 3 seedlings were analyzed per genotype. Cells analyzed are the same as in Fig 2G. P-values were calculated using nonparametric statistical analysis (Kruskal–Wallis test); * p-value < 0.05; **** p-value < 0.0001. (PDF) [file pgen.1011637.s003.pdf]

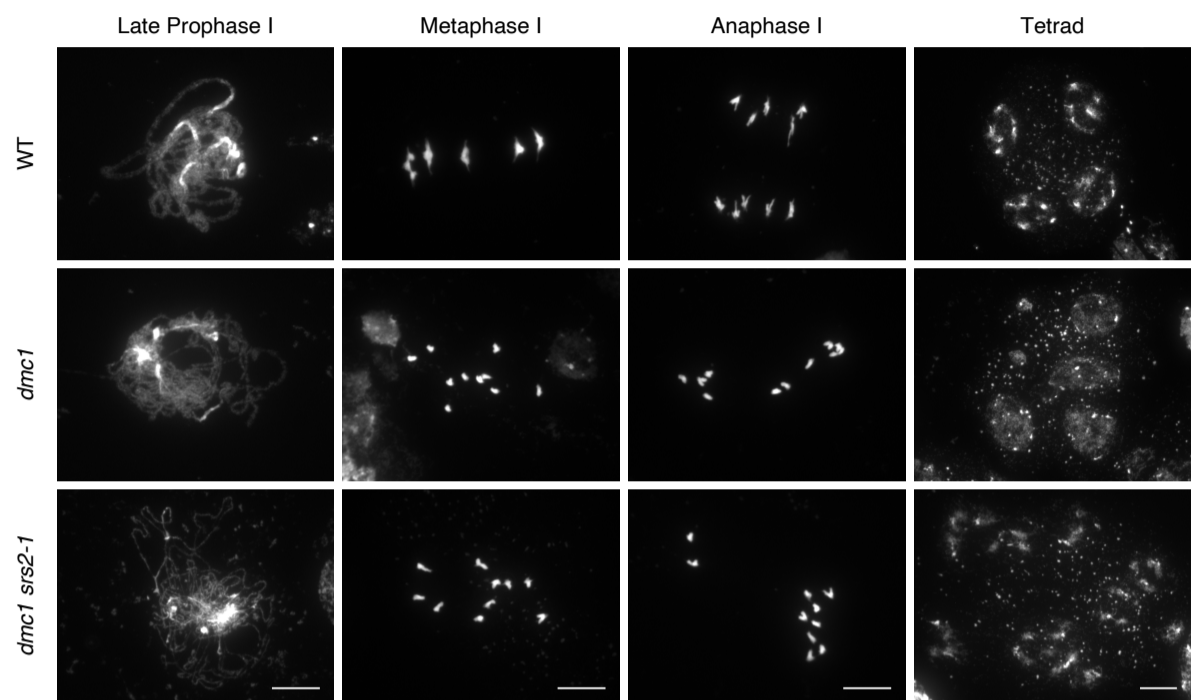

Supplement: S4 Fig — Meiotic progression of male meiocytes stained with DAPI in WT, dmc1, and dmc1 srs2–1 plants. Absence of DMC1 leads to asynapsis (late Prophase I) and lack of inter-homologue CO. Intact univalents are thus observed at Metaphase I owing to DSB repair by RAD51, most probably using sister chromatids. Univalents then segregate randomly at Anaphase I and ultimately this produces unbalanced Tetrads. A similar meiotic phenotype is observed in dmc1 srs2 mutant. Scale bar: 10 µm. (PDF) [file pgen.1011637.s004.pdf]

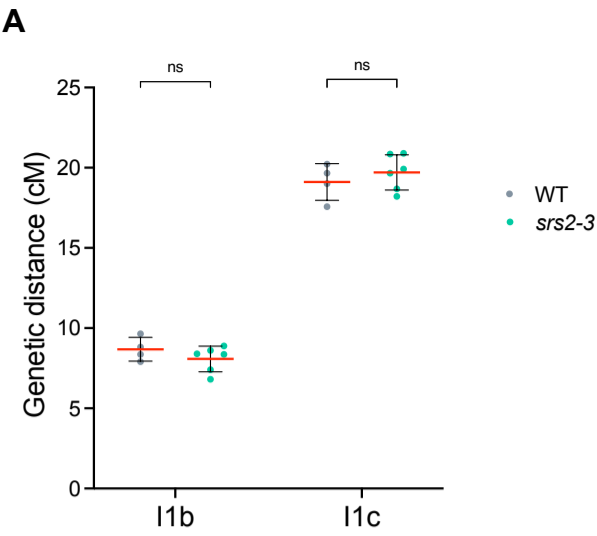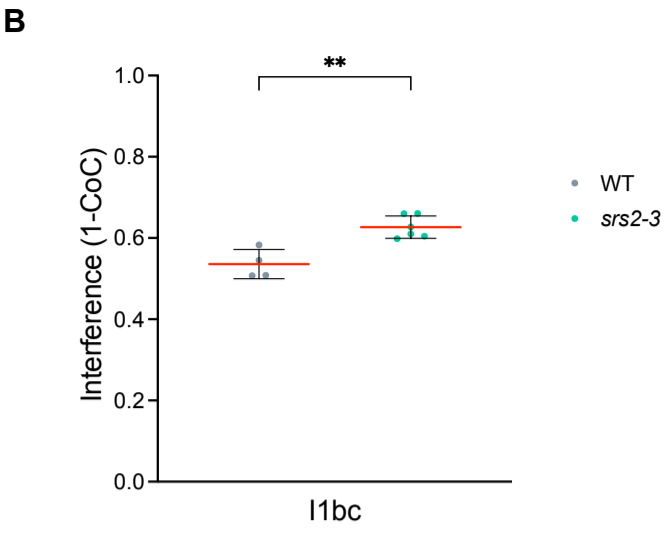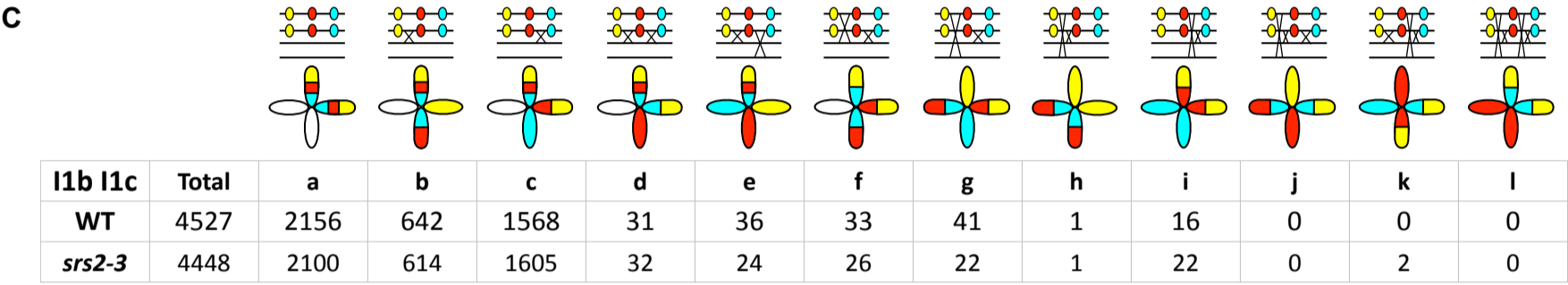

Supplement: S5 Fig — (A) CO frequency of WT and srs2–3 mutant within I1bc interval represented as the genetic distance (cM). Each dot represents one plant, with 400–800 tetrads analyzed per plant. Mean is presented as a red bar. (B) Interference within I1bc interval of WT and srs2–3 plants. Each dot represents one plant. Mean is presented as a red bar. Statistical analysis was performed using Z-test. (PDF) [file pgen.1011637.s005.pdf]

**A**

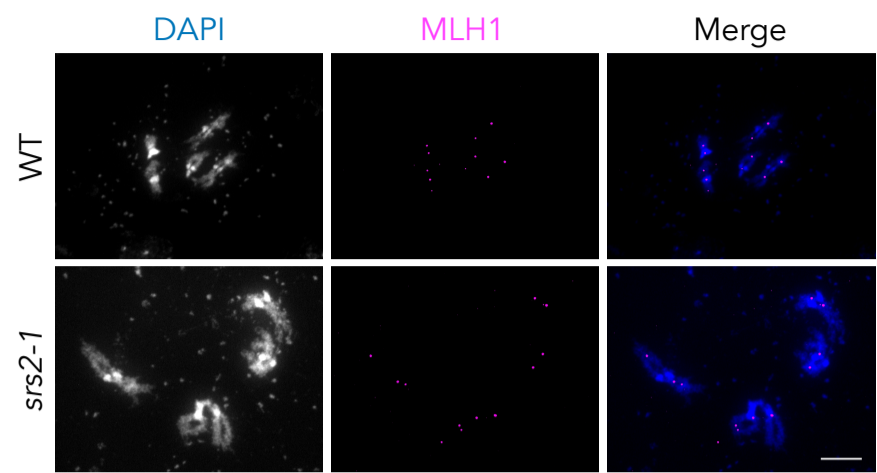

**B**

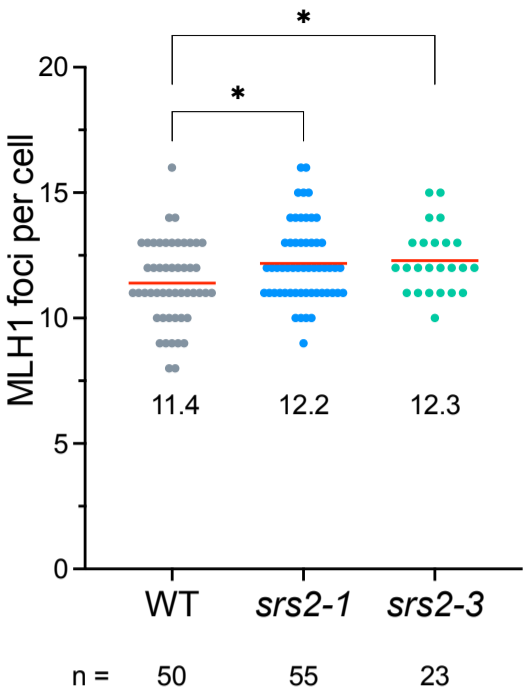

Supplement: S6 Fig — (A) Representative images of MLH1 immunostaining on diakinesis-staged male meiocytes in WT and srs2–1 mutant. Scale bar: 10 µm. (B) Number of MLH1 foci per cell in srs2–1 and srs2–3, with n indicating the number of cells analyzed. Data are represented as mean (red line), with each dot representing one individual cell. Statistical analysis was performed using Mann-Whitney test. * p-value < 0.05. (PDF) [file pgen.1011637.s006.pdf]
