# Supplementary material for: SRS2 is required for MUS81-dependent CO formation in zmm mutants
Source: PLoS Genet. 2025 Aug 7;21(8):e1011637. doi: 10.1371/journal.pgen.1011637 (PMC12349706; doi:10.1371/journal.pgen.1011637)
Supplement: S3 Table — (DOCX) [file pgen.1011637.s009.docx]

**S3 Table. List of primers used for characterizing *Atsrs2* T-DNA mutants**

| Allele | Primer Name | Sequence 5' - 3' | Use |
| --- | --- | --- | --- |
| *SRS2* | A | AAGAAGAATGGAAGCAAAACT | Genotyping  *srs2-1*/ RT-PCR |
|  | B | TGAATCAAATCCTGAACTGTC | Genotyping  *srs2-1*/ RT-PCR |
|  | C | TATGCCAAGGATCTATCAAAA | RT-PCR |
|  | D | TTGTACTGCATTGTGCTAGTG | RT-PCR |
|  | E | GAAGAGACTCCTACCATTGCT | RT-PCR |
|  | F | AGATTTTCCTATGCGTTCTCT | RT-PCR |
|  | G | ATTTCTGGTACCTTCAAGAGG | Genotyping  *srs2-3*/ RT-PCR |
|  | H | TTAAGCAGTTTTCCTCCATC | Genotyping  *srs2-3*/ RT-PCR |
|  | I | TGACTTTGATGATTCCATTTT | Genotyping *srs2-2* |
|  | J | ATAACACCCCCTAAGTGACAT | Genotyping *srs2-2* |
| GABI-Kat  T-DNA | K | TGGACGTGAATGTAGACACGTCG | Genotyping GABI  T-DNA left border |
| SALK  T-DNA (Lba1) | L | TGGTTCACGTAGTGGGCCATCG | Genotyping SALK  T-DNA left border |
| *ACTIN 2* | ACT2-F | CCTTGTACGCCAGTGGTCG | RT-PCR |
|  | ACT2-R | CTCGGCCTTGGAGATCCAC | RT-PCR |
