# Supplementary material for: SRS2 is required for MUS81-dependent CO formation in zmm mutants
Source: PLoS Genet. 2025 Aug 7;21(8):e1011637. doi: 10.1371/journal.pgen.1011637 (PMC12349706; doi:10.1371/journal.pgen.1011637)
Supplement: Attachment — Submitted filename: response to reviewers.docx [file pgen.1011637.s012.docx]

Dear Dr. Da Ines,

Thank you for submitting your manuscript to PLOS Genetics. After careful consideration, we feel that it has merit once appropriate modifications and possibly additions of new data are made. Therefore, we invite you to submit a revised version of the manuscript that addresses the points raised during the review process.

Please include the following items when submitting your revised manuscript:

* A rebuttal letter that responds to each point raised by the editor and reviewer(s). You should upload this letter as a separate file labeled 'Response to Reviewers'. This file does not need to include responses to any formatting updates and technical items listed in the 'Journal Requirements' section below.

* A marked-up copy of your manuscript that highlights changes made to the original version. You should upload this as a separate file labeled 'Revised Manuscript with Track Changes'.

* An unmarked version of your revised paper without tracked changes. You should upload this as a separate file labeled 'Manuscript'.

If you would like to make changes to your financial disclosure, competing interests statement, or data availability statement, please make these updates within the submission form at the time of resubmission. Guidelines for resubmitting your figure files are available below the reviewer comments at the end of this letter.

We look forward to receiving your revised manuscript.

Kind regards,

Additional Editor Comments (if provided):

All three reviewers are enthusiastic about your work. To strengthen your argument regarding the function of SRS2, we kindly ask you to carefully read the reviewers' comments and address them by explaining in a more concise manner, providing additional information to further validate your claims, or rephrasing or toning down some statements where appropriate. If you need any clarification or assistance, don’t hesitate to contact me. I’m here to help ensure the review process goes smoothly.

Dear Editor,

We are very pleased with the positive feedback of all three reviewers to our manuscript and thank them for their valuable suggestions. We have responded to all reviewers’ comments and incorporated new data into the revised manuscript. Specifically, we have added an analysis of SRS2 AlphaFold model in the revised Figure 1, reanalyzed the MMC sensitivity of *srs2* mutants (revised Figure 2C), and included an analysis of pollen viability (revised Figure 3B). We have also toned down or rephrased some statements where appropriate, as suggested by the reviewers.

Our responses to each point raised by the reviewers and the changes we have made in the manuscript are detailed below (The reviewers' comments are in blue 11-point type and our responses in black 11-point type).

We thank the reviewers for their comments, which have helped us to significantly strengthen our manuscript, and we now hope it will be suitable for publication in PLOS Genetics.

Sincerely,

Reviewer's Responses to Questions

Reviewer #1: The authors present their findings on the role of the SRS2 helicase in both somatic and meiotic cells in Arabidopsis thaliana. Interestingly, they show a different role of SRS2 in somatic cells than what has been showed in yeast. The authors also study the role or SRS2 in meiotic cells, where they show its involvement in both class I and class II crossover regulation. Particularly, their data shows that SRS2 is needed for the formation of class II crossovers, when, to date, only few actors have been shown to be involved in this pathway.

We thank the reviewer for her/his kind comments on our manuscript.

Major comments:

1. Changes in the strength of interference are not enough to claim that interference (between class I COs) is changed. The addition of class II crossover in the mix of class I can lead to apparent changes in interference strength without affecting the interference mechanism per se. For instance, Berchowitz et al 2007 show that in mus81 mutant interference increases. Conversely, in mutants with elevated class II crossover count, interference is decreased (Crismani et al., 2012, Girard et al., 2014 etc…). It is very likely that the genetic interference measured in srs2 could be a consequence of increasing the class II pathway, and not that SRS2 is a positive effector of class I COs. In the Discussion, this is actually how the authors phrase it (lines 388 and 411), but in the results, the wording can lead to confusion (lines 255-274). If the authors wish to claim that srs2 is responsible for an increase in genetic interference (between class I COs), then they should measure the CoC in a srs2 mus81 double mutant. The expectation is that if there is a direct effect of SRS2 in interference, the CoC would be higher in the double mutant compared to the single mus81 mutant. Otherwise, given the epistasis between SRS2 and MUS81 the increase in genetic interference should be discussed as being most likely due to a decrease in class II crossovers. This is actually a good argument for the decrease of class II COs that the author could capitalize on (in addition to fig 6 results). I would therefore strongly recommend showing the interference measure after the data with mus81 (fig 6) to avoid any confusion.

We thank the reviewer for this insightful comment. We agree that the wording in the Results section (previously lines 255-274) could misleadingly suggest a direct effect of SRS2 on genetic interference. As stated in the Discussion, we favor the hypothesis that the observed increase in genetic interference in the *srs2* mutant is an indirect consequence of the reduction in Class II COs. We have now revised this paragraph (now lines 298-312), as well as in the following paragraph (lines 315-318) to clarify this point and ensure consistency with the Discussion. We do however believe it is still appropriate to present the interference data at this stage, as it associates with the use of the FTL lines and is essential for the subsequent rationale assessing class II COs.

2. Figure 7: The defects shown in the paper are very moderate. Therefore, in the model in Fig. 7, the ++ and - - effects in the srs2 mutant could be toned down (to + and -, indicating a slight increase and a slight decrease). Same for the thickness of the arrows, the data show an increase of 1 class I CO, which does not deserve such a strong arrow.

We thank the reviewer for this helpful suggestion. We have modified the figure (now Figure 6) accordingly to better reflect the moderate nature of the observed phenotype. Specifically, we have reduced the thickness of the arrows. In response to this comment and to a related point raised by Reviewer 3, we have also added the average number of Class I and Class II COs (as measured in this study) for both WT and *srs2* mutant directly into the figure.

- Figure 7: Additionally, considering their model, it feels like the authors are claiming that SRS2 would act directly on the loading/dynamic of RAD51/DMC1. Based on data from yeast, this is plausible. However, the presented data does not support this hypothesis more than an alternative one where the turnover of RAD51 foci is altered due to some defects in repair. If the authors want to indeed claim a direct role of SRS2 on RAD51 filament, they should at least quantify the RAD51 foci during meiosis and not only in somatic cells, where only a slight increase was shown. Otherwise, they should remain vague in the figure.

We fully agree with the reviewer. The slight increase in RAD51 foci in somatic cells is not sufficient to claim that SRS2 acts directly on the loading or dynamics of RAD51, especially as concerns meiosis. As noted by the reviewer, the modest increase in RAD51 foci could equally well be due to minor defects/delays in the repair pathway, and we do not expect to observe a difference during meiosis (particularly given the absence of fertility or pollen defects). Accordingly, and in addition to our changes mentioned above, we have removed the speculation concerning a putative effect on RAD51 nucleofilament dynamics, as this is not supported by conclusive data, especially in the context of meiosis.

Minor comments/manuscript organization:

1. Generally, it would be nice to have conclusions as Figure titles (2 / 3 / 4), or at least making the titles a little bit more informative

We have revised the figure titles to make them more informative, as suggested.

2. Figure 1 could be in supplementary figures as it is not a major result but simply a verification of T-DNA insertions. Perhaps it would be more interesting to show the (predicted) structures of yeast Srs2 and plant SRS2 proteins, with the conserved and non-conserved residues, with a particular focus on their putative functionality (as discussed in the Discussion section)

We thank the reviewer for this valuable suggestion. The figure showing the verification of T-DNA insertions is now Figure S2. Additionally, we have included a new Figure 1 showing AlphaFold structure prediction of AtSRS2 and a comparison and superimposition with budding yeast ScSrs2. This clearly shows a strong conservation of the helicase domains.

3. In Fig2 and Fig3 the authors show that the srs2 mutants are not more susceptible to DNA damage, which, interestingly, is different from what has been shown in yeast. Is SRS2 expressed in somatic cells in Arabidopsis? Are there paralogs of srs2 in the Arabidopsis genome? Could there be some redundancy?

SRS2 is indeed expressed in somatic tissues, in addition to its expression in meiocytes. BLAST analysis did not reveal the existence of other UvrD-type helicase in Arabidopsis genome (Blanck et al., 2009, and our analysis). This is now clearly stated in the manuscript (lines 208-211).

Furthermore, as the reviewer suggested, functional redundancy with other helicases could explain the lack of increased sensitivity to induced DNA damage observed in *srs2* mutants. While we have tested potential redundancy with RAD54, RECQ4A, MUS81, and RTEL1, no additive effects were observed in the corresponding double mutants (Figure 2C). Although we cannot exclude redundancy with other untested helicases, our results show no major role for SRS2 in somatic recombination.

4. In Fig3C, the data layout makes it difficult to see any differences between the genotypes, and Fig3D is redundant (same type of data). Perhaps it could be enough to show that RAD51 focus number increases upon treatment with MMC in srs2 mutants using only Fig3D (and keep 3C for supp data).

We have simplified the former Figure 3 by removing Fig3D plot, and by merging the former panels 3A, 3B and 3D with Figure 2. These are now included in the updated Figure 2. Additionally, the former Figure 3C has been moved to the new Figure S3.

5. I would recommend fusing Fig2 and 3 (it would become figure 1) to showcase the differences in requirement for SRS2 in mediating somatic repair in response to DNA damaging agents. This could be accompanied, if the authors chose to, by the fusion of section 2 and 3 of the text. The main message is that Srs2 is dispensable for DNA repair and RAD51-dependent recombination in somatic cells, unlike in yeast, which is interesting.

We thank the reviewer for this valuable suggestion. We have now merged Figure 2 and Figure 3, as well as the corresponding sections in the manuscript (now lines 201-261). The revised Figure 2 now shows that, unlike in yeast, Arabidopsis SRS2 is not essential for RAD51-dependent recombination in somatic cells and does not significantly affect RAD51 nucleofilament formation following DNA damage induction.

6. Fig5 srs2-3-containing data could be relegated to a supplementary figure. However, FigS3 should absolutely be together with l1bc interval data for srs2-2 mutant. This would better reflect the reality of the data (slight change in interference strength)

We thank the reviewer for this valuable suggestion. We have moved *srs2-3* FTL data to supplementary Figure S5, while I2fg data have been incorporated into the main Figure 4. Additionally, FTL counting data have been included into the revised Figure 4.

7. Fig5: Why showing C/D + E/F? Is the difference between the two wild-type controls in the two experiments (11 foci in D vs 8 co-foci in E) something we should take into account? Perhaps merging MLH1 data and ignore the Hei10, or just show the number of co-localised MLH1/HEI10 foci?

There is indeed a slight difference between the mean number of MLH1 and MLH1/HEI10 foci, which can be explained by more stringent counting in co-immunolocalization. We believe MLH1/HEI10 co-immunolocalization is more robust as it better excludes false-positive foci that may occur when immunolocalizing MLH1 alone. Accordingly, we have modified the section of the manuscript discussing Class I CO numbers (lines 318-326) and removed MLH1 data from the new Figure 4. The MLH1 data (formerly Figure 5C-D) have been moved to the supplementary Figure S6.

Typos:

- Line 80: “Srs2 (Suppressor of RAD Six-screen mutant 2)” and remove (Suppressor of RAD Six-screen mutant 2) from line 85

done

- Line 94: Remove parenthesis in “SDSA” and add “Synthesis-Dependent Strand Annealing”

done

- Line 106: remove “single-stranded DNA” since ssDNA has been defined before

done

- Line 127: in plants

done

- Line 131: In vitro

done

- Line 155: a homolog of the yeast Srs2 was found

done

- Line 159: Remove “(SRS2)”

done

- Line 188: Remove “strand”

done

- Line 222-224: The deviation does not match the deviation written on Fig3C

We thank the reviewer for pointing out this error. The figure presents standard deviation (SD), while we incorrectly reported SEM in the manuscript. We have now revised the manuscript to report SD, as presented in the figure (lines 240-241).

- Line 244-246: Split in two sentences

Done (now lines 276-278)

- Line 294: Replace “In accordance with expectation,” by “As expected,”

Done (now line 331)

- In Materials and Methods some antibody references are missing (e.g., a-RAD51 in line 483; Rabbit MLH1 and Chicken HEI10 in line 534, donkey a-rabbit Cy3 and goat a-Chicken Alexa 488 in lines 536-537; guinea pig a-ASY1, rabbit a-ZYP1 in line 558; donkey a-rat Cy3 and goat a-guinea pig alexa 488 in lines 560-561)

References for the antibodies have now been added in the Materials & Methods section (lines 586-587, 589-590, 612, 615-616).

- Fig1A legend: In line 848-849 remove “Triangles (purple, blue, or green triangles).” (not a sentence)

Done (now in Figure S2; line 1059).

- Fig1C legend: In line 857 remove ACT and use just Actin RT+ as shown in the figure.

Done (now in Figure S2; line 1067).

- Figure2B legend: Missing total number of seedlings analyzed. Meaning of p-value **** and * missing

The total number of seedlings analyzed has now been added in the revised Figure 2 caption (line 952). We have also added in the caption the meaning of **** and * p-values (lines 953-954).

- Figure2C legend: Missing total number of seedlings analyzed.

This has now been added as above (line 957).

- Figure3D legend: Missing meaning of “ns” (non-significant)

The reviewer is likely referring to Figure 2D. The meaning of “ns”, non-significant is now stated in the figure legend (line 966).

- Fig2C missing which T-DNA insertion srs2 mutant was used in the double mutants

We have revised Figure 2C to indicate that the T-DNA insertion mutant used to generate the double mutant lines is *srs2-1*.

- Fig5B: Scale should range from 0 to 0.8 and not 0.4 to 0.8

The revised Figure 4B now also includes I2fg. The scale of the figure has thus been corrected to range from 0 to 1.

- Throughout the figures, please homogenize the statistics to plot the mean + SD and not the mean + SEM in all plots.

All plots have been homogenized to display mean + SD.

Reviewer #2: The manuscript by Petiot et al. describes their investigations into the in vivo role of the SRS2 homolog from Arabidopsis thaliana, a helicase that plays an important role in homologous recombination by regulating the strand invasion process in yeast. In plants (Arabidopsis), a previous in vitro/biochemical study demonstrated that SRS2 has a role in homologous recombination processes, but its function in plants remains unknown. In the current manuscript, these authors utilized multiple null alleles of SRS2 to characterize this gene in Arabidopsis. They conducted detailed experiments to demonstrate that, in contrast to yeast studies, AtSRS2 is dispensable for somatic homologous recombination, meiosis progression, and fertility in Arabidopsis. However, through an impressive set of cytological observations, quantifications, and recombination assays using fluorescent-tagged lines, the authors show that AtSRS2 plays a pro- and anti-crossover role in meiosis. The following are my comments on the manuscript.

We thank the reviewer for her/his positive comments on our manuscript.

The authors use the fluorescent-tagged lines/markers (FTLs) in a tetrad mutant background to score for recombinants. This well-established assay is, in general, very informative. However, the authors should provide more information on the marker positions on the chromosomes and in the context of centromeres (e.g., provide a schematic with physical distances). Centromeres are known to negatively affect recombination, and this is not discussed concerning markers on studied chromosome 1 or 2. Are those loci studied on the chromosome potentially affected by centromeres?

We thank the reviewer for the insightful comment. In response, we have added a schematic to the revised Figure 4 showing the positions of the fluorescent markers and the physical distances. The two intervals tested (I1bc and I2fg) in our study are located on the arms chromosome 1 and 2, respectively, and are well described in the literature. We have added a statement (now line 294-297) to the Results section to clarify that these intervals are not located in or near centromeres.

One of the major claims of the authors regarding the role of SRS2 in recombination is based on the results from chromosome 1. The findings on chromosome 2 are not very convincing in the context of interference, given that the authors state in line 271, “.... not statistically significant, likely due to the very short size of the I2fg interval, which in practice limits the detection of a sufficiently large number of double CO events. If the authors believe that the short size of the I2fg interval limits the occurrence of significant double CO events, then they might consider the FTLs on other chromosomes or possibly collect data by sequencing multiple sets of tetrad progenies.

We thank the reviewer for highlighting this important limitation. As also noted by Reviewer 1, we hypothesized that the observed increase in genetic interference within the tested intervals results from a reduction in Class II COs, which is accompanied by a slight increase in the number of Class I COs, similar to what was observed in the *mus81* mutant (Berchowitz et al. 2007). Since Class II COs are not sensitive to interference, their reduction could lead to an apparent increase in interference strength due to a shift in the overall Class I to Class II ratio in these intervals. We agree with the reviewer that the use of FTL intervals alone is not sufficient to definitively conclude a change in interference, and we believe that adding another interval would not significantly strengthen this claim. We have tested two intervals I1bc and I2fg for srs2-1 and both show an increase in genetic interference. Although the increase is not statistically significant, the tetrad analysis data (now included in revised Figure 4) clearly show a reduction in the number of double COs in the srs2-1 mutants the I2fg interval (6 double COs in srs2-1 vs. 12 in the WT). In response to the reviewer comment (and to reviewer 1 related point above) we have toned down our interpretation where appropriate and revised Figure 4 accordingly. The revised Figure 4 now includes both I1bc and I2fg data in the *srs2-1* mutant (including tetrad counts). Additionally, I1bc data in the *srs2-3* mutant are now presented in Figure S5, as well as all FTL raw data and calculations in Table S1.

Recombination and crossover frequencies differ between male and female meiosis. Do the authors think the SRS2 might have a differential role in these processes?

This is indeed an important point. As mentioned by the reviewer, recombination frequencies and CO rates vary significantly between male and female meiosis. However, whether SRS2 plays a similar, or different role in female meiosis has not been addressed in this study. All our experiments and observations (cytology, pollen viability, immunolocalization, FTL analysis) have been performed in the context of male meiosis. We are thus not able to further speculate on the role of SRS2 in female meiosis.

Given the authors' expertise in immunolocalizing crucial proteins involved in Class I and Class II crossovers, the localization of AtSRS2 in meiosis and mitosis is explicitly missing. Can the authors demonstrate that it is indeed physically part of the recombination process either by colocalization or showing its interaction with one or more Arabidopsis recombination machinery components in vivo?

We thank the reviewer for raising this point. Unfortunately, no SRS2 antibody is currently available, which prevents us from immunolocalizing SRS2 in somatic and meiotic cells. Regarding protein-protein interaction, we tried to evaluate potential interactions between SRS2, RAD51 and DMC1 using Yeast Two Hybrid (Y2H, but unfortunately, strong autoactivation in our constructs prevented us from drawing any conclusions and prevented going further with this approach. We have also explored in silico interaction predictions using AlphaFold. However, SRS2 appears very unstructured (see also Figure 1), resulting in relatively poor predictions (even when using budding yeast SRS2 and RAD51, which are known to interact). At present, the only supported claim we can make is that SRS2 transcripts are expressed in both somatic and meiotic cells. This is explicitly stated in the manuscript (lines 208-210 and 411-412).

Regarding SRS2's pro-CO role, is there a way to demonstrate that the lack of SRS2 results in rechanneling the NCO to the ZMM pathway? E.g., overexpression of AtSRS2 in meiosis?

We thank the reviewer for the valuable comment. Studies in yeast (e.g. Sasanuma et al. 2013) have indeed shown that overexpression of Srs2 affects meiotic recombination by removing Rad51 from recombination intermediates, suggesting a precise level of SRS2 is required for the regulation of RAD51 nucleofilament formation during meiosis, and potentially for COs/NCOs formation. In contrast, DMC1 filaments appear to be unaffected. This work is explicitly considered in the discussion (lines 415-418). It is indeed an interesting hypothesis that we will test. However, as Arabidopsis SRS2 overexpression lines are currently not available, this is probably beyond the scope of this paper and will require significant additional work.

Although the authors raise concerns about the lack of conservation of ScSrs2 Y775 (V963 in Arabidopsis) related to “potential functional divergence or loss ” in both the results and discussion sections of this manuscript, its relevance to the proposed role (pro and anti CO?) remains unclear from the discussion. Additionally, the authors cite a previous study on SRS2 in Physcomitrella. A comparison of AtSRS2 vs. PpSRS2 in the context of ScSRS2 amino acid conservation (Y775) and other sites could be more insightful.

The lack of conservation of ScSrs2 Y775 (corresponding to V963 in Arabidopsis) may indeed suggest a functional divergence in SRS2 activity. Specifically, Y775 has been shown to be required for D-loop disruption in Yeast (Meir et al.2023). We initially used this observation to highlight possible differences between the yeast and Arabidopsis proteins. However, we agree that it is not directly relevant to the proposed role of AtSRS2 in crossover control. As also noted by reviewer 1, we favor the interpretation that AtSRS2 primarily acts to promote Class II crossovers, rather than playing a dual role with anti-Class I, which is supported by the redirection of Class II crossovers to the Class I pathway in the srs2 mutant background. We have revised the manuscript accordingly to clarify this point and to emphasize that our speculation about the lack of conservation of ScSrs2 Y775 concerns specifically the D-loop activity of SRS2 (lines 176-182 and 357-361). Moreover, as suggested by the reviewer, we have included PpSRS2 protein sequence in our protein sequence comparison in Figure S1. This analysis shows that the amino acid at the corresponding position in PpSR2 (V1120) is also not conserved, while other residues remain conserved. This further support our hypothesis of a functional divergence in D-loop processing among plant SRS2 homologs.

Incorporating the tetrad analysis data into the main figure (with all types of crossovers, especially double crossovers relevant to interference, highlighted) will allow readers to better appreciate the subtle yet intriguing role of AtSRS2 in meiosis.

Following the reviewer’s suggestion, we have included tetrad analysis data (with counts and schematics for each CO type) to the revised Figure 4. This better illustrates the reduction in double COs.

Figure 3: Rad51 foci per nuclei. Do you have any thoughts on why the majority of cells do not exhibit RAD51 foci after MMC treatment? However, when they are observed, most RAD51 foci-positive nuclei show 10 or more signals. Is a specific cell cycle stage more vulnerable to MMC treatment than others? What threshold size was used to call a given fluorescent signal as a RAD51 signal with respect to any potential background signals?

DSB repair by RAD51 occurs primarily during S and G2 phases of the cell-cycle. In Arabidopsis root apex, most cells are outside of S/G2 phases therefore, only a few cells are expected to exhibit RAD51 foci, even after MMC treatment. However, when foci are present, the number of foci is rather high due to the relatively high MMC dose and extended treatment duration. The number of foci is correlated with MMC dose and treatment time and this is well-documented in the literature.

As our images were acquired using Z-stacks, we set a standard foci size of 0.3 µm in diameter, and 0.5 µm z-axis elongation (This is now specified in materials and methods section, line 539-540). We found that this standard size fit the majority of RAD51 foci. To distinguish true foci from background signal, we manually set a fluorescence intensity threshold based on the “Intensity Sum of Squares” in the EGFP (RAD51) channel. This approach allowed the software to reliably detect foci above the defined threshold while excluding background noise.

Figures 3A, 3C, 3D, and lines 218-219: I see a few RAD51 foci in the charts, so please change the statement accordingly. My understanding is that there is a background level of somatic HR that is RAD51 but may not be efficiently captured.

We thank the reviewer for pointing this out. There is indeed a very low level of spontaneous breaks and RAD51 foci are occasionally observed even without treatment. We have revised the sentence accordingly to read “As expected, very few RAD51 foci were detected…. “ (now line 250).

Figure 4: Fertility in Arabidopsis can be measured in various ways, but it is often not done or reported accurately. The number of seeds per silique or the observation of meiotic abnormalities through cytology seem to be reasonable measures, but they lack stringency when the events are relatively rare yet biologically relevant (not spontaneous) compared to WT. One additional estimate on the female side could be the count of unfertilized ovules in matured or developing siliques (represented by tiny, dried, whitish ovules) and aborting ovules after fertilization (brownish ovules in matured or developing siliques). On the male side, pollen viability can be assessed through staining, such as using Alexander staining on mature, intact anthers. If the abnormalites are relatively scarce in the srs2 mutant background, counting the chiasmata in 45 or 66 meiocyte spreads, as reported in this manuscript, may not serve as an ideal assay.

We thank the reviewer for this valuable suggestion. To further support our fertility data (seeds per silique, meiotic progression and chiasma number), we performed Alexander staining on approximately 3000 pollen grains per genotype and found no difference between the *srs2* mutants and the wild-type. These new results are now presented in panel B of the revised Figure 3, and mentioned line 265.

Lines 159-160: What do the authors mean by all the amino acids recently identified as essential? This is not entirely clear.

We thank the reviewer for his comment. We mean the amino acid residues recently identified, using AlphaFold, as being in contact with DNA. These residues were further shown to compromise Srs2 activity (Meir et al., 2023). We have clarified this point in the manuscript (lines 170-175).

Figure 2B: Any thoughts of a significant increase in sensitivity to MMC 40uM concentration by WT strains compared to mutants?

This was indeed something we noticed initially. However, it was not reproducible in subsequent experiments (see e.g. Figure 2C) and we thus concluded that the difference (less than 0.5 leaf) falls within the range of experimental error.

Figure 2 C: Which srs2 mutant alleles were used in single and double mutant studies? Only one allele is marked (srs2-1), while the others are not identified. Why do the number of true leaves in the control (WT) and other mutants/double mutants exceed those in Figure 2B? Why is there this discrepancy?

All double mutants shown in Figure 2 were generated using *srs2-1* mutant. Figure 2C has been revised accordingly to reflect this information. Regarding the discrepancy in the number of true leaves between Figure 2B and 2C, we believe it to be due to differences in scoring methods. The two experiments were conducted independently, at different times, and leaf counts were estimated by different experimenters using distinct methods: assessment with a magnifying class in Figure 2B and a binocular microscope observation in Figure 2C. To ensure consistency and comparability, we have now reproduced the experiment involving the double mutants (Figure 2C) using magnifying class to assess leaf number. This is now presented in revised Figure 2C.

Figure 2D; Include some representative GUS staining images and the interrupted GUS construct’s schematic to aid readers.

Representative image of GUS staining and the construct’s schematic have been added to the revised Figure 2 to facilitate readers understanding (Figure 2D).

Expand SDSA at its first mention in the manuscript

Done (now line 93).

Lines 278-280: sentences appear convoluted; paraphrasing would be more helpful to readers

We agree that (former) lines 278-280 could be confusing for the reader. We have thus rephrased the sentence to clarify the message (now lines 315-318).

Figure 1 Legend: fix the repetition of triangles

As also suggested by Reviewer 1, we removed the repetition of triangles in legend of the revised (now) Figure S2 legend (line 1059).

To aid readers, label the amino acids mentioned in lines 161-166 with specific coordinates on Figure S1.

We have now labelled the amino acids on both the new Figure 1 (see response to reviewer 1) and the revised Figure S1. We have also added the sequence of Physcomitrella SRS2.

Reviewer #3: Petiot et al seek to understand the in vivo role of SRS2 helicase in Arabidopsis meiotic recombination. AtSRS2 has been shown to possess helicase activity in vitro in prior studies. The authors in this work find a dispensable role for AtSRS2 in mitotitc recombination while a shift in the balance between class I COs and class II COs is observed in the absence of SRS2. The data presented shows a role for SRS2 similar to that of MUS81. The manuscript overall is well written with the data presented as elegant figures.

We thank the reviewer for her/his kind comments on our manuscript.

Major questions/comments

Why the authors performed the non-parametric Kruskal-Wallis test is not clear? Are the data analyzed not normally distributed? In an exception to this, two other tests were performed in Fig. 4 and Fig. 6. Is there an explanation to this? This will affect a lot of the data interpretation in this study.

We thank the reviewer for pointing out this inconsistency. For all statistical analysis, we first assessed the normality of data distributions using the D’Agostino–Pearson test. When comparing two independent datasets, we used an unpaired Student’s t-test if both followed a normal distribution, or a Mann–Whitney test otherwise. For comparisons involving more than two datasets, we performed a Kruskal–Wallis test followed by Dunn’s post hoc test for multiple comparisons. When comparing datasets with multiple experimental conditions (e.g., Fig. 2. treated vs. untreated seedlings), we used a two-way ANOVA. For the statistical analysis of FTL data, we used a Z-test. We have now included a “Statistical analyses” section in the Materials and Methods (lines 648- 655), and homogenized the statistical methods used across all figures accordingly.

An important question is about the proposed “dual role” for SRS2 in Arabidopsis. The idea of a “dual role” for SRS2 is not very convincing. The data presented shows that Atsrs2 mutant has reduced class II CO (Fig) and an increased class I CO and genetic interference (Fig). This data is similar to the observation for mus81 mutant (lines 408-409). Further, the authors show that mus81 is epistatic to srs2 and indeed the triple mutant zip4srs2mus81 is not different from zip4srs2 or zip4mus81. All of these point to the fact that SRS2 functions similarly and probably together with MUS81 in promoting/balancing class II CO. If this is the case, would the authors also argue that MUS81 has a “dual role”?

We completely agree with the reviewer that MUS81 should not be considered a having a “dual role”, and agree that this phrasing may be confusing for the reader. In the Discussion section, we propose that SRS2 could stabilize some recombination intermediates for their resolution mediated by MUS81 (lines 457-459). In the absence of SRS2, these intermediates are likely redirected into the ZMM pathway, thus increasing Class I COs while decreasing Class II COs. To improve clarity and avoid confusion, we have modified the title of the manuscript (line 3-4) and removed all mention to a “dual role” throughout the text. Please also see our response to a similar comment raised by reviewer 1 above.

The idea of shifting the balance between Class I COs and Class II Cos for both SRS2 and MUS81 is more convincing.

We agree and this point is now emphasized in the manuscript.

The model presented in Fig .7 could be improved by adding more details. Currently, the visuals don’t convey a lot. For example,” what is the “++” supposed to mean in the right panel next to the cartoon of RAD51 and DMC1? Adding details to the graphic will convey the message better. Additionally, can the authors include an estimate of the number of class I CO and class II CO in the right panel from the data obtained in this work?

We thank the reviewer for her/his suggestion. We have revised Figure 6 with estimates of CO numbers based on our results in both WT and *srs2* mutant. As also suggested by Reviewer 1, we have reduced the thickness of the Class I CO arrow for the WT, to improve clarity.

Minor suggestions:

Cite figures in “discussion”

done

Specific details:

Line 94: explain SDSA at first mention

Done (line 93)

Are there other UvrD-type DNA helicases in Arabidopsis, such as an FBH1 like (lines 115-121)?

We thank the reviewer for this comment. SRS2 appears to be the only UvrD-type helicase encoded in the Arabidopsis genome (lines 207-211). However, the Arabidopsis genome contains a homolog of RTEL1, which is proposed to act as a functional homologof Srs2 in mammals. However, our srs2 rtel1 double mutant did not exhibit hypersensitivity to MMC. This point is addressed in the discussion (lines 373-380).

Line 462: expand MMC and provide catalog number, if possible

Done and reference has been added to the Materials and Methods section (line 502-503).

Line 468: how were the lines generated? Briefly, include the crossing scheme in a few words.

The lines were generated by crossing the IU.GUS-8 line with *srs2-*1 and *srs2-3* mutants. The crossing scheme has been added to the Materials and Methods section (lines 509-511)

Lines 481-482: expand PFA. What is the molar concentration corresponding to “1x”?

PFA has been expanded and the molar concentration corresponding to 1X has been added to the Materials and Methods section (lines 524-525).

Line 483: what is the source of anti-RAD51?

Th reference for the anti-RAD51 antibody has been added to the Materials and Methods section (line 527).

Line 513: for how long?

The duration of inflorescence fixation in Carnoy’s fixative has been added to the Materials and Methods section (line 564).

Lines 848-849: “Triangles…” is redundant

The redundancy has been removed from (now) Figure S2 legend.

Line 869: data are from how many experiments is missing for panel C. This information is provided for panel B.

The total number of seedlings analyzed in Figure 2C has been added in the revised Figure 2 legend (line 952).

Line 873: similar comment as above.

The total number of seedlings analyzed in Figure 2D has been added in the revised Figure 2 legend (line 964).

Line 957: Giving species abbreviation before the protein would be helpful, such as ScSrs2, etc.

Done (lines 1043-1044)

Lines 962-963: what do the authors mean by “Srs2 activities”? It is counterintuitive that the essential amino acids are not conserved but the Arabidopsis SRS2 has in vitro helicase activity. Could it be written in a different way?

As written in the manuscript, all but one of the “essential” amino acids are conserved in Arabidopsis (and Moss). These amino acids have been shown to be instrumental for the recombinogenic activities of Srs2 in Yeast (Meir et al., 2023). Only Srs2 Y775, essential for D-loop activity of ScSrs2 is not conserved in Arabidopsis (and Moss). We propose that this may indicate a functional divergence of plant SRS2 with respect to D-loop activity. To avoid any confusion, we have modified “activities” to “functions” (line 1051).
